# Circadian regulation of metabolic, cell division, and cation transport promoters in the gastrointestinal bacterium *Klebsiella aerogenes*

**DOI:** 10.3389/fmicb.2023.1181756

**Published:** 2023-07-05

**Authors:** Kinga B. Graniczkowska, Jiffin K. Paulose, Vincent M. Cassone

**Affiliations:** ^1^Department of Biology, University of Kentucky, Lexington, KY, United States; ^2^Division of Human Genetics, Cincinnati Children’s Hospital, Cincinnati, OH, United States

**Keywords:** circadian, melatonin, gastrointestinal, microbiome, *Klebsiella*

## Abstract

**Introduction:**

All eukaryotes and at least some prokaryotes express the capacity to anticipate and adapt to daily changes of light and temperature in their environments. These circadian programs are fundamental features of many forms of life. Cyanobacteria were the first prokaryotes to have demonstrated circadian gene expression. Recently, a circadian rhythm was also discovered in an unrelated bacterium, *Klebsiella aerogenes*, a human gut commensal and nosocomial pathogen.

**Methods:**

Here we characterize new clock-controlled genes with spatial differences in expression using a bacterial luciferase reporter. These include dephospho-coenzyme A kinase (*coaE*), manganese transporter, H-dependent (*mntH*) and a gene identified as filamenting temperature-sensitive mutant Z (*ftsZ*).

**Results and Discussion:**

The data show that all three reporter constructs exhibited circadian variation, although only PmntH::luxCDABE reporter strains were synchronized by melatonin. Additionally, we show that *K. aerogenes* divides rhythmically in vitro and that these bacteria may alternate between exponential and stationary cells. Together, these findings provide a deeper understanding of *K. aerogenes*.

## Introduction

The gut commensal bacterium *Klebsiella (née Enterobacter) aerogenes* is a gram-negative, indole-negative member of the family *Enterobacteriaceae.* While it is commensal in the gastrointestinal tract, it is known to cause severe nosocomial infections in hospitalized patients. Most of these arise as urinary tract infections, causing 6%–17% of all nosocomial infections, and likely originate from patient’s gastrointestinal tracts, hands of hospital staff, unsanitary medical equipment and blood products (Podschun and Ullmann, 1998; Surve and Bagde, 2011).

Previous research in our laboratory has shown that cultures of *K. aerogenes* are sensitive to the pineal and gut hormone melatonin in that macrocolonies on semi-solid media increase in size in response to exposure to this hormone in a specific, dose-dependent fashion (Paulose et al., 2016). Indoleamines tryptophan, 5-hydroxytryptamine, and N-acetylserotonin had, in contrast to melatonin, no effect on culture size in concentrations ranging from 1 pM to 1 nM. Transcriptomic analysis of liquid cultures of *K. aerogenes* revealed that 1 nM melatonin affected several physiological components, including metal cation transport and binding, stress response, fimbrial assembly and biofilm related genes. The pattern of differential expression was largely restricted to cultures in exponential growth phase (Graniczkowska et al., 2022).

Macrocolonies on semi-solid media exhibited concentric rings that were expressed rhythmically with a period of approximately 25 h, suggesting a circadian rhythm (Paulose et al., 2016; Graniczkowska and Cassone, 2021). To better visualize this rhythmicity, cultures of *K. aerogenes* were transformed to express a reporter construct in which the promoter of flagellar stator protein *motA* was fused to bacterial luciferase (Paulose et al., 2016). Bacterial luciferase is a self-sufficient biochemical system in which all substrates can be recycled and formed by a group of enzymes from the *lux* operon (Tinikul et al., 2020). The DNA sequences encoding the peptides from the bioluminescent system are named the *lux* genes, and they are located on the *luxCDABE* operon from *Photorhabdus luminescens*. Bacterial luciferase is a commonly employed real-time reporter of gene expression used to study circadian rhythmicity in cyanobacteria (Kondo et al., 1993) and *B. subtilis* (Eelderink-Chen et al., 2021).

Cultures transformed with *PmotA::luxCDABE* expressed circadian patterns of bioluminescence with periods (τ) ranging from 22 to 28 h. These periods did not significantly vary in ambient temperatures (T_A_) ranging from 27°C to 40°C, indicating that the clock underlying *K. aerogenes*’ rhythmicity is temperature compensated (Paulose et al., 2016). In the absence of melatonin, individual culture plates exhibited circadian patterns that were not synchronized among culture plates and therefore had phases that were not coincident. However, in the presence of 1 nM melatonin, cultures exhibited surprisingly synchronous cycles. The mechanism for this synchrony is not known at this stage.

While the circadian clock of *K. aerogenes* is temperature-compensated, appearing to be insensitive to ambient temperature, cycles of bioluminescence can be entrained to cycles of ambient temperature (T_A_) that are similar in amplitude to the daily changes in body temperature (T_B_) (Paulose et al., 2019). Cycles of T_A_ of 1°C (35°C–36°C) or 3°C (34°C–37°C) in amplitude and of varying periods (T-cycles) systematically entrained bioluminescence rhythms. T-cycles of 22, 24, and 28 h. synchronized rhythmic patterns of *PmotA::luxCDABE* bioluminescence such that higher levels of bioluminescence predominated during the higher temperature of the cycle. However, when released into constant temperature, rhythms persisted with a phase directly related to the phase of the last high temperature. These data showed that the clock underlying *K. aerogenes* rhythmicity could theoretically entrain to subtle changes of T_B_ within the gastrointestinal system. Similar rhythms have been shown in liver tissue explants and cultured fibroblasts entrained to cycles of T_A_ (Brown et al., 2002; Brown and Azzi, 2013). The *K. aerogenes* bioluminescence rhythm has a very high amplitude during the temperature entrainment (Paulose et al., 2019), and upon releasing to free running conditions, the rhythm persists for at least 4 days. However, the amplitude of this signal greatly decreases in constant conditions.

Since the previous studies relied exclusively on determining expression patterns of *motA,* the data may only reflect an effect on bacterial motility. To test rhythmicity of other gene expression patterns, bacteria were transformed with a plasmid containing a luciferase cassette driven by other selected promoter regions from the *K. aerogenes* genome. We chose three candidate genes, *coaE*, *ftsZ*, and *mntH*, based on their function or sequence homology. *CoaE* gene encodes dephospho-coenzyme A kinase, which we previously showed shares some homology at the protein level with the cyanobacterial clock protein KaiC (Paulose et al., 2016). However, domain analysis indicated that it is not functionally similar (Paulose et al., 2016). CoaE is also the final enzyme involved in the biosynthesis of the important cofactor coenzyme A (Mishra et al., 2001; Obmolova et al., 2001). Manganese transport, H-dependent (MntH) protein, as the name suggests, is involved in transport of Mn (II) ions (Jurková, 2009). In the enterobacteria, this locus is subjected to complex regulation in response to manganese, iron, and reactive oxygen species. Transport of divalent metal ions into the cell by MntH is dependent on the membrane potential and extracellular pH (Jurková, 2009). MntH was identified during the search for sequence similarity to human melatonin receptor binding sites among gut microbiota encoded peptides (Paulose et al., 2016). FtsZ is a cell division protein; it is ubiquitous in bacteria and can also be found in chloroplasts (Mori and Johnson, 2001). FtsZ is a GTPase with a structure similar to tubulin. It forms ring-shaped polymers at the site of cell division along with other proteins such as FtsA, ZipA, and ZapA. FtsZ was selected as an indicator of bacterial cell division. Additionally, this gene is rhythmically expressed with a circadian period in *Synechococcus elongatus* (Mori and Johnson, 2001). The present study demonstrates rhythmic expression of these three clock-controlled genes, as well as addressing potential mechanisms of rhythm damping.

## Methods

### Reporter construction

The previously described plasmid *PmotA::luxCDABE* (Paulose et al., 2016) was isolated and digested with the EcoRI restriction enzyme to replace the promoter sequences. The promoter region of *coaE*, *mntH*, *or ftsZ* were amplified using polymerase chain reaction (PCR) and a high-fidelity polymerase (Quanta Biosciences, Gaithersburg, MD) and subsequently ligated, using ElectroLigase^®^ (NEB, Hitchin, United Kingdom), to a backbone of the digested plasmid. Then, wild type electrocompetent *K. aerogenes* cells were electroporated with one of the reporter plasmids. New constructs were confirmed by PCR and Sanger sequencing.

### Bioluminescence monitoring

Semisolid (0.67% agar) eosin methylene blue (EMB) agar (1 mL/well), containing 0 nM or 1 nM melatonin, was placed in the black 24-well microplate with a clear bottom (VisiPlate-24 Black, Perkin Elmer, Waltham, MA, United States). Medium was allowed to solidify and dry completely under a biosafety cabinet. Subsequently, 1 μL of overnight culture was spotted into the center of each well and allowed to dry. Wells were sealed with 18×18 mm cover glasses with sterile vacuum grease and placed into the *in vivo* imaging system (IVIS Spectrum, Perkin Elmer, Waltham, MA, United States) for 96 h. Bioluminescence measurements were taken every hour using a 1 s exposure time. Living Image Software (IVIS Spectrum, Perkin Elmer, Waltham, MA, United States) was used for bioluminescence analysis for Average Radiance (p/s/cm^2^/sr) quantification within selected ROIs (regions of interest, Supplementary Figure S1). The second method we used for bioluminescence monitoring implemented a photon counter Lumicycle 32 (Lumicycle, Actimetrics, IL). Bacteria expressing *coaE::luxCDABE* reporter were inoculated in the center of 35 mm plates filled with semisolid EMB. Plates were covered with 40 mm cover glasses by sterile vacuum grease and placed into a Lumicycle automated photomultiplier-based bioluminescence recorder. Each sample was counted for 70 s on a rotating platform. Raw bioluminescence baselines were subtracted using a 24-h running average via Lumicycle Analysis software (Actimetrics, IL).

### Bacteria number quantification

Bacteria were cultivated and recorded using a cooled CCD camera [Perkin Elmer *In Vivo* Imaging System (IVIS)] as described above. At the same time, the number of colony-forming units (CFUs) responsible for the bioluminescence signal was evaluated. CFUs were assessed in the initial overnight culture, used for the inoculation, and from selected wells from the 24-well plate during each timepoint. Briefly, every 6 h, a plate was removed from the IVIS, and the content of 2 wells was collected and placed in two separate sterile tubes with 10 mL water. Tubes were shaken vigorously until the semisolid agar completely broke down, and bacteria were transferred into the solution. This suspension was serially diluted and plated on agar plates for the CFU assessment. CFUs were counted after 12 h of incubation.

### Plasmid stability

We implemented a 24-well microplate for the macrocolony cultivation; 1 mL of semisolid (0.67% agar) eosin methylene blue (EMB) agar (HiMedia Laboratories, Mumbai, India) was placed in each well. Bacteria collected from three wells per timepoint were serially diluted in 24 h. intervals for 6 days. For the subsequent CFU count, we used a “dribble” plate serial dilution technique. Dribble plating is a method for reducing the number of plates needed for counting bacteria. It is a modified standard protocol, where 10 μL of 8 serial dilutions of bacteria are applied simultaneously on the edge of 100 × 100 mm square agar plates using a multichannel pipet and allowed to run down (dribble) the length of the plate parallel to each other (Lionello et al., 2019). Each sample was plated on solid EMB agar with or without tetracycline to allow assessment of plasmid loss. After overnight incubation, CFUs were counted manually.

### RNA sequencing

Planktonic cultures of *K. aerogenes* grown in tryptic soy broth (TSB) media were collected during the exponential and stationary phase. Total RNA was extracted using hot phenol method. Ribosomal rRNA was depleted prior to sequencing implementing the Ribo-Zero™ Magnetic Kit (Illumina, Inc.). A library for strand-specific transcriptome sequencing was prepared using NEBNext^®^ Ultra™ Directional RNA Library Prep Kit for Illumina^®^ (NEB, United States) following the manufacturer’s recommendations, and index codes were added to each sample. RNA sequencing was performed on an Illumina Hiseq platform. Clean reads were aligned to the reference genome (Shin et al., 2012) using Bowtie2–2.2.3 (Langmead et al., 2009). The number of reads that mapped to each gene was evaluated by HTSeq v0.6.1. Then the expected number of Fragments Per Kilobase of transcript sequence per Millions (FPKM) base pairs sequenced, of each transcript was calculated (Langmead et al., 2009). A more detailed protocol was previously published (Graniczkowska et al., 2022).

### Analytical statistics

Daily patterns of gene expression were analyzed using the MetaCycle package 1.2.0 (Wu et al., 2016) in RStudio version 1.3.1, with JTK_CYCLE and Lomb-Scargle methods and the circadian minimum-maximum period range of 22–28 h. We used the “meta2d” function (ARS, JTK, LS) for all reporters. Based on the noisiness of these data and because MetaCycle was designed for microarray data, we also ran a less stringent cosinor function with harmonics: CircWave (Hut; https://www.euclock.org/results/item/circ-wave.html), with the same range of possible periods. CFUs data were also analyzed using CircWave. CircWave assumes a 24 h period and applies harmonics to the basic sinusoidal function. These attributes allowed us to use the software to determine whether the data were rhythmic for each day of the 2-day sampling as well as providing centers of gravity to determine acrophase for each day.

## Results

### Rhythmicity and spatial distribution of *coaE*, *ftsZ* and *mntH* reporters

**Figure 1:**
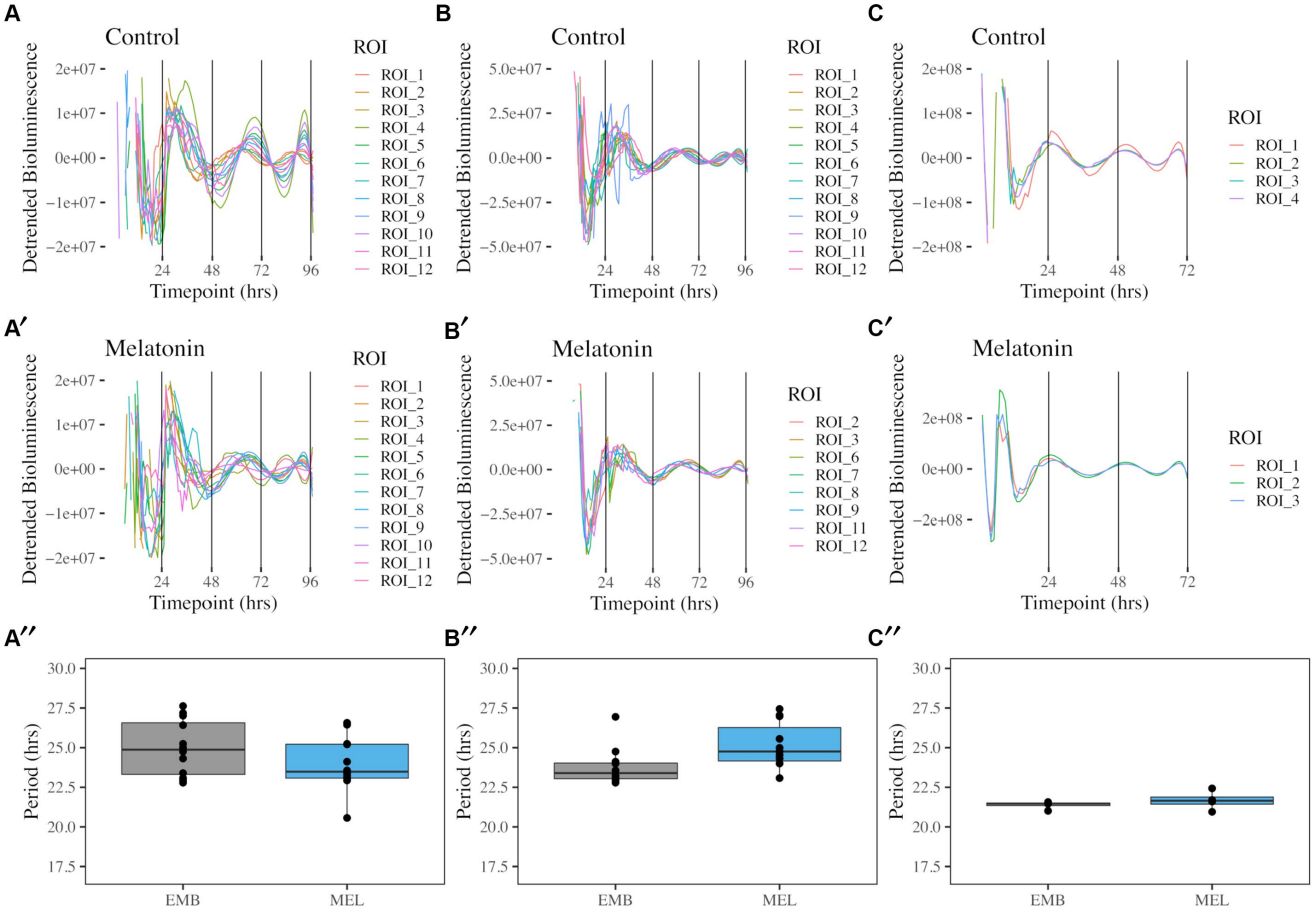
Free running rhythms and periods of *PcoaE-*, *PftsZ-*, and *PmntH::luxCDABE* reporters taken from the center of each culture well. Detrended records of bioluminescence from *PcoaE::luxCDABE* in standard media [**(A)**, *n* = 12] and media supplemented with 1 nM melatonin [**(A′)**, *n* = 12]. Detrended bioluminescence traces from *PftsZ::luxCDABE* in standard media [**(B)**, *n* = 12] and 1 nM melatonin-supplemented media [**(B′)**, *n* = 11]. Detrended bioluminescence traces from *PmntH::luxCDABE* in standard media [**(C)**, *n* = 4] and 1 nM melatonin-supplemented media [**(C′)**, *n* = 4]. Period analysis for *PcoaE::luxCDABE* **(A″)**, *PftsZ::luxCDABE* **(B″)**, and *PmntH::luxCDABE* **(C″)** comparing cultures in the absence (EMB) or presence (MEL) of melatonin.

**Figure 2:**
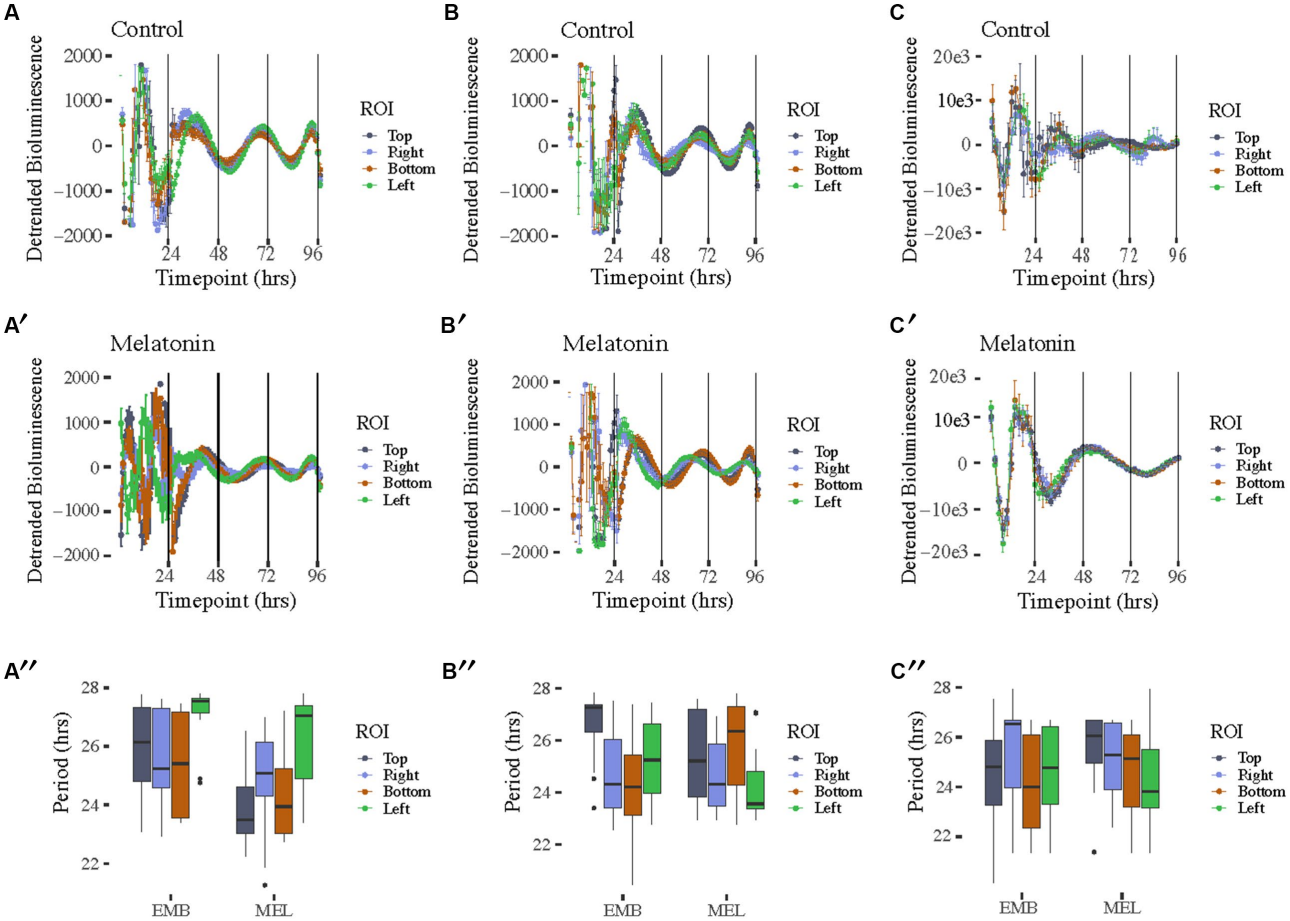
Free running rhythms and periods of *PcoaE-*, *PftsZ-*, and *PmntH::luxCDABE* reporters taken from the each of four peripheral ROIs in each culture well. Detrended records of bioluminescence from *PcoaE::luxCDABE* in standard media [**(A)**, *n* = 12 for top, right, and left, *n* = 10 for Bottom] and media supplemented with 1 nM melatonin [**(A′)**, *n* = 12 for top, right, bottom, and left]. Detrended bioluminescence traces from *PftsZ::luxCDABE* in standard media [**(B)**, *n* = 12 for top, bottom, and left, *n* = 8 for right] and 1 nM melatonin-supplemented media [**(B′)**, *n* = 12 for top, right, bottom, and left]. Detrended bioluminescence traces from *PmntH::luxCDABE* in standard media [**(C)**, *n* = 4 for top, *n* = 14 for right, *n* = 8 for bottom, *n* = 9 for left] and 1 nM melatonin-supplemented media [**(C′)**, *n* = 13 for top, *n* = 10 for right, *n* = 7 for bottom, *n* = 12 for left]. Period analysis for *PcoaE::luxCDABE* **(A″)**, *PftsZ::luxCDABE* **(B″)**, and *PmntH::luxCDABE* **(C″)** comparing cultures in the absence (EMB) or presence (MEL) of melatonin for each peripheral ROI.

The free-running rhythms of *coaE*, *ftsZ* and *mntH* promoter activity in *K. aerogenes* were identified by implementing bacterial luciferase as a real-time reporter of gene expression. Circadian rhythms were detected for all three reporters, independent of melatonin presence in the growth media or quantified region of interest (ROI) (Figures 1, 2). A free-running rhythm in the above-mentioned reporters’ activity occurred when bacteria were incubated at 37°C. The period calculated over a 96-h window following media inoculation was 24.97 ± 0.50 h for *PcoaE::luxCDABE* and 23.95 ± 0.48 h for the same reporter when melatonin was in the media (Figures 1A,A′). Statistical analyses revealed no difference in period length when semisolid agar contained 1 nM melatonin (Figure 1A″, ANOVA with Tukey’s post-hoc test, *p* = 0.525). The free running period of *PftsZ::luxCDABE* was 23.79 ± 0.33 (Figure 1B), and 25.06 ± 0.46 with melatonin (Figure 1B′). No significant difference was observed in *PftsZ::luxCDABE* recordings in the presence or absence of melatonin (Figure 1B″, *p* = 0.311). The free running period of *PmntH::luxCDABE* was 21.38 ± 0.12, and 21.66 ± 0.30 h in the absence (Figure 1C) or presence (Figure 1C′) of melatonin over 72 h. No significant difference was observed between the two media (Figure 1C″, *p* = 1.000). Free-running rhythms in promoter activity of *coaE*, *ftsZ*, and *mntH* were detected in 100% of cultures when period was calculated over a 72-h window. However, during the fourth day of the experiment, we observed a drastic decrease in the number of rhythmic colonies, which was associated with the loss of bioluminescence signal in some wells (Supplementary Videos S1, S2).

The bioluminescence signal captured with the IVIS allowed us to also characterize the spatial distribution of the bioluminescence signal in the motility assay (Figure 2). A high amplitude signal of bioluminescence was recorded in constant temperature for 4 days with a free-running period ranging from 22 to 28 h, however, each day of the experiment the bioluminescence signal decreased. These results were comparable with our previous reports of data recorded with the Lumicycle [with τ (tau) = 24.5 ± 0.5 h] (Paulose et al., 2016). The bioluminescence signal originated from the center, where bacteria were inoculated and with time proceeded toward the periphery (Supplementary Videos S1, S2). Interestingly, we observed spatial differences among *coaE, ftsZ*, and *mntH* reporters. From the second day of the experiment *coaE* bioluminescence signal covered a larger area of the well compared with *ftsZ* and *mntH*. This could be a result of three-dimensional differences in the expression of these promoters within the macrocolony. Additionally, we observed morphological differences in the appearance of the macrocolonies, which only became apparent after 3 days of culture. Four ROIs around the initial centrally placed ROI were analyzed to determine if macrocolony shape influenced rhythmicity (Supplementary Figure S1). As previously mentioned, all promoter constructs showed rhythms in both media conditions (Figure 2). Periods from all ROIs showed similar ranges between 22 and 28 h with no significant differences found between complementary ROIs in control EMB and melatonin-treated media (Figures 2A″,B″,C″). Of the three reporters, *PmntH::luxCDABE* oscillations were the most asynchronous in the absence of melatonin (Figure 2C) and 24-h periodicity was lost within 48 h, even in the presence of melatonin (Figure 2C′), despite a 10-fold higher peak amplitude. Implementing the luciferase reporter, driven by a promoter cloned from the host’s genome, on the plasmid introduces additional copies of that promoter in the cell. The plasmid we used in this experiment contains the medium-copy-number p15A ori (origin of replication), which means that each bacterium contains around 10 copies of this plasmid (Botella et al., 2012; Fan et al., 2014). Depending on the sigma factor which binds to the promoter before the gene is transcribed, additional copies of this promoter may recruit limited copies of transcriptional factors resulting in disrupted gene expression. This could explain differences in the macrocolony appearance, which may also be responsible for the spatial differences in bioluminescence.

To validate our bioluminescence imaging results, we also implemented Lumicycle photomultiplier, even though this method does not allow for the spatial differentiation of signal. Bioluminescence signal recorded in this experiment indicated robust circadian patterns in 70% of cultures and the period calculated over a 48-h window following media inoculation was 23.54 ± 2.72 h for *PcoaE::luxCDABE* (Supplementary Figure S2).

### Decrease of bioluminescence rhythm amplitude

**Figure 3:**
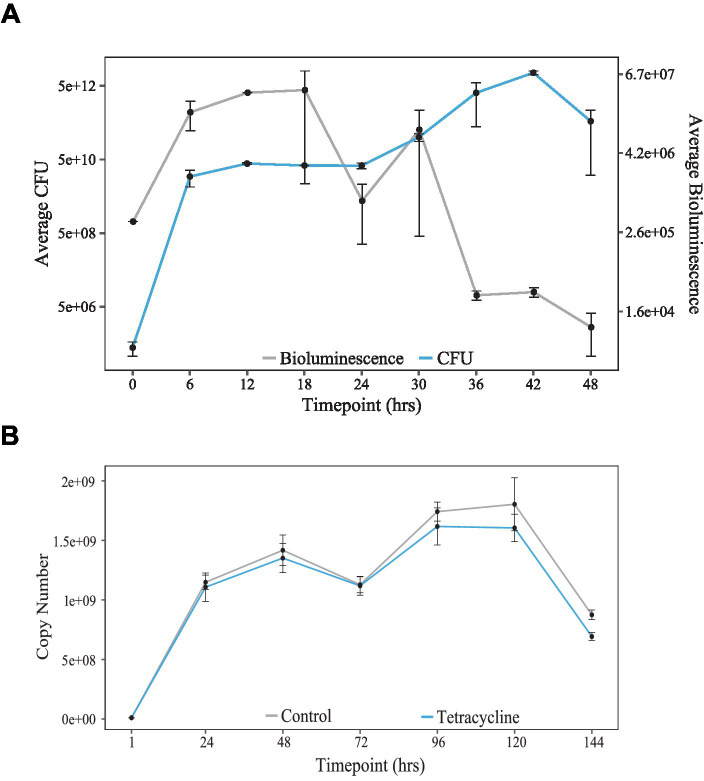
Population growth in *K. aerogenes*. **(A)** Copy number stability over 144 h in cultures incubated with Tetracycline (blue) or in standard media (grey) [adapted from Graniczkowska and Cassone (2021)]. **(B)** Culture growth, as seen by colony forming units (CFUs, blue) compared to bioluminescence (grey) over 48-h.

As briefly mentioned earlier, the *K. aerogenes* bioluminescence signal decreases in amplitude after release to a constant temperature. A potential explanation is that these microorganisms are losing the plasmid carrying the luciferase reporter. We had tested the stability of the plasmid previously (Graniczkowska and Cassone, 2021), and plasmid loss was not significant during 6 days of the motility assay (Figure 3A). Therefore, we excluded it as a reason for the signal decrease. Other explanations include that the bacteria are either entering the stationary growth or even dying in these culture conditions. To test the numbers of live bacteria in our culture settings, we developed a novel protocol, allowing for the evaluation of live bacteria responsible for the bioluminescence production at various circadian times. Briefly, bacteria were cultivated on 24-well plates in constant temperature. At the same time, we monitored the bioluminescence signal to test the correlation between the number of live bacteria and the amount of light produced. Content of 2 wells per timepoint was removed from the plate and serially diluted in 6 h. intervals for 2 days. Fractions of bacterial suspension were plated and incubated for subsequent colony-forming units (CFUs) enumeration. During the initial 12 h of the experiment, both the number of CFUs and the amount of bioluminescence increased rapidly (Figure 3B). After the first peak, bacteria did not increase their numbers probably because they entered the stationary phase and the bioluminescence signal decreased. After 24 h post-inoculation, bacteria started to divide rapidly again, and the second smaller peak of bioluminescence was observed at timepoint 30. However, even though the amount of CFUs was still rising until hour 46, the bioluminescence signal did not follow the same pattern. Statistical analysis using CircWave revealed rhythmicity in the bioluminescence intensity and colony-forming unit numbers.

Next, we wanted to test if the observed rhythms were due to periodic switching to stationary growth during the motility assay. To do so, we performed an RNA-Seq experiment on planktonic cultures of *K. aerogenes,* and we sequenced exponential and stationary cells. The growth conditions had to be altered to be able to collect exponential and stationary grown bacteria, which is not possible on the plate. Total RNA isolated from the liquid cultures of *K. aerogenes* was used for the library preparation and sequenced [discussed in detail in Graniczkowska et al. (2022)]. The results were analyzed to obtain and characterize differential gene expression (DEG) between two growth stages. Analysis revealed that *ftsZ*, and *mntH* are differentially expressed between growth stages. However, *motA* and *coaE* are not differentially expressed (Table 1).

**Table 1:** Expression of candidate genes during exponential and stationary growth.

Gene	Product	Readcount expo	Readcount stat	Log2fold change	*p*_adj_	DEG
*coaE*	Dephospho-CoA kinase	575.24	491.22	0.22	0.07318	False
*ftsZ*	Cell division GTPase	5541.97	9440.88	−0.76	4.26E-12	True
*mntH*	Manganese transport protein	86.02	552.38	−2.68	9.14E-32	True
*motA*	Flagellar motor protein	17.84	28.14	−0.65	0.12696	False

## Discussion

Based on these results, we conclude that *ftsZ*, *coaE*, and *mntH* are clock-controlled genes in *Klebsiella aerogenes*. They are expressed in a circadian fashion. However, the amplitude of bioluminescence decreases with each day of cultivation. We observed a clear spatial distribution of the bioluminescence signal originating from the inoculation and spreading to the periphery in the form of concentric rings. Therefore, a method that allows focus on the specific area with a homogenous signal; IVIS is ideal for quantifying this expression. When comparing two bioluminescence datasets recorded with two different methods (Lumicycle vs. IVIS) we observed various waveforms. It is crucial to point out that Lumicycle data were quantified for the entire colony, and in case of IVIS we were able to focus on the smaller area of the center of the colony. Nevertheless, no matter the method we always observe a daily drop in the amplitude of the signal.

Additionally, *K. aerogenes* on a population level divides rhythmically. Most bacteria exhibit cell division times that are much more frequent than is the 24-h. day, making circadian patterns in bacteria a multi-generational phenomenon (Mori et al., 1996). At the same time, even rapidly growing *S. elongatus* shows rhythmicity of cell division with the generation time of 8–12 h long. In contrast, during exponential growth, the generation time of *K. aerogenes* is only 30 min (Kelly and Rahn, 1932). However, a circadian pattern of gene expression at a population level is still apparent. In cyanobacteria, the clock of daughter cells is in phase with the clock of their mother cells. This population rhythmicity is achieved by inheritance of KaiC in specific phosphorylated state corresponding to the defined circadian time (Golden et al., 2007). At this point the molecular driver of *K. aerogenes* clock is still unknown but it is possible that it is also driven by posttranslational modifications similarly to cyanobacteria.

Cyanobacteria divide during the daytime (LD) or subjective day (LL) and stop dividing in early subjective night. This behavior is driven by a KaiC-dependent clock (Mori et al., 1996), which specifies the time of the day at which cell division is permitted. We report here a circadian pattern of *K. aerogenes* CFUs over a 2-day window (Figure 3B). Additionally, morphological changes in form of concentric rings (Graniczkowska and Cassone, 2021) in the appearance of *K. aerogenes* microcolony suggest that these bacteria regulate its cell division in circadian fashion comparable to cyanobacteria (Mori et al., 1996). We hypothesize that concentric rings are created by circadian control of cell division and/or transition between exponential and stationary bacteria. Electron microscopy on dissected microcolonies would answer the question whether these bacteria also alternate between exponential and stationary growth phase since these two stages of growth are associated with different cell shapes similar to *E. coli* (Siegele and Kolter, 1992). Exponential *K. aerogenes* is rod shaped, whereas stationary cells are spherical (Graniczkowska, 2021). Additionally, RNA sequencing results suggest that transitions between exponential and stationary growth may contribute to the rhythmicity of gene expression, since *ftsZ*, and *mntH* are differentially expressed when comparing exponential and stationary cells (Table 1). On the other hand, *motA* and *coaE* are also rhythmically expressed but are not affected by the growth stage; therefore, other factors must be responsible for the circadian pattern of its expression. We would like to point out that the RNA-Seq was performed on bacteria collected from planktonic cultures (Graniczkowska et al., 2022), but rhythmicity of luciferase reports was investigated in motility assay on semisolid agar. Additionally, the RNA-Seq samples were collected from bacteria grown in TSB and reporters’ analyses were performed using EMB media. Both the growth conditions and variations in nutrients available in the media may have impact on the gene expression pattern; thus, it is difficult to make direct comparisons. Nevertheless, alternating zones of exponential and stationary bacteria, corresponding to the visual variations in the morphology of the microcolony (Graniczkowska and Cassone, 2021) is a potential explanation of spatial differences in gene expression.

Furthermore, circadian rhythmicity of *K. aerogenes* is synchronized by melatonin (Paulose and Cassone, 2016; Graniczkowska and Cassone, 2021). The concentration of gut melatonin measured in mouse stool follows circadian pattern (Paulose et al., 2019), suggesting that melatonin, could be another *zeitgeber* for this bacterium, in addition to T_B_. In the above-mentioned experiment, *coaE* bioluminescence rhythmicity differs significantly when bacteria were incubated with 0 nM vs. 1 nM melatonin. However, *coaE* was not identified among differentially expressed genes sensitive to melatonin (Graniczkowska et al., 2022). Once again, these experiments were performed in different growth conditions; thus, direct comparisons are not possible. Nevertheless, these results suggest that the regulation of *coaE* expression is complex and depends on nutrient availability and/or growth substrate. In contrast, *mntH* expression *was* synchronized by the presence of melatonin in the media, particularly when observing ROIs outside of the center of the wells. The sensitivity of this transporter to reactive oxygen species may underlie its sensitivity to melatonin, as melatonin is a potent antioxidant (Reiter et al., 2016).

*K. aerogenes,* as a human gut commensal bacterium, lives in rhythmic conditions of fluctuating core body temperature, daily changes in concentration of host-delivered antimicrobial peptides, gut mucosal antibodies, nutrient availability, and shifting composition of commensal gut bacteria community (Thaiss et al., 2014; Paulose and Cassone, 2016; Parkar et al., 2019; Paulose et al., 2019; Graniczkowska, 2021). Therefore, it is understandable that this bacterium over a course of coevolution with humans and other commensal hosts adapted to its host’s circadian rhythmicity and developed its own mechanism to anticipate diurnal changes in its environment to gain advantage over the competition in a very competitive niche of human gastrointestinal tract.

The fact that a commensal gut bacterium has its own endogenous clock adds to the hierarchical organization of human circadian rhythms. The understanding of this phenomenon will provide important insights into the complexity of microbiome-host interactions and human health. We (Paulose and Cassone, 2016) have proposed that circadian organization resembles a meta-organism of circadian clocks within circadian clocks: the circadian oscillators in the brain entrain circadian oscillators in the periphery (Hoogerwerf, 2006; Malloy et al., 2012), such as in the intestines, which subsequently affects bacterial circadian clocks. While this paper provides evidence of rhythmicity in three additional clock-controlled genes and our recent findings demonstrating a daily pattern of *K. aerogenes* motility (Paulose et al., 2016), this observation is in accordance with the metagenomic analysis reported by Thaiss et al. (2016), indicating daily oscillation in the functional composition of microbial genes. More specifically, upregulation of motility genes in the resting phase of the host. Daily patterns of flagella biogenesis can be explained by the need to seek nutrient sources and mucus penetration (Hoogerwerf, 2006; Parkar et al., 2019). Therefore, *K. aerogenes* expresses motility genes, such as *motA*, rhythmically to maximize the benefits of interaction with the host and optimize its own fitness to gain advantage over other members of gut microbiota.

It is interesting to note that *motA, ftsZ, coaE,* and *mntH* represent very different parts of the genome and differing metabolic (KEGG; Kyoto Encyclopedia of Genes and Genomes) pathways. Yet, they all express circadian rhythmicity. This suggests that the circadian clock within this organism must broadly regulate gene expression. Further, it is also interesting to note that while all of these genes are expressed rhythmically, the circadian rhythms of two of these genes, *motA* (Paulose et al., 2016) and *mntH,* are synchronized in the presence of melatonin, while the other two, *coaE* and *ftsZ*, are not. It will be important to identify the mechanisms of melatonin action, and these studies are currently underway.

In summary, we characterized three new clock-controlled genes with spatial differences in expression and investigated potential causes for the decrease in bioluminescence amplitude. While it is unlikely that the decrease is due to plasmid loss, there may be other mechanisms (e.g., decreased O_2_ concentration). Additionally, we showed that *K. aerogenes* divides rhythmically *in vitro* and that these bacteria may alternate between exponential and stationary cells. However, more evidence is required. Further assessment of rhythmicity of other genes is needed and, more importantly, identification of its core oscillator is crucial at this point to better understand the circadian rhythmicity of this human gut commensal.

## Data availability statement

The datasets presented in this study can be found in online repositories. The names of the repository/repositories and accession number(s) can be accessed at the Gene Expression Omnibus [GEO, NCBI, NIH] Accession number: GSE172068.

## Author contributions

KG, JP, and VC planned the studies, wrote and edited the manuscript. KG conducted the research. JP analyzed the data. All authors contributed to the article and approved the submitted version.

## Funding

These studies were funded by NIH R01 GM118541-01.

## Conflict of interest

The authors declare that the research was conducted in the absence of any commercial or financial relationships that could be construed as a potential conflict of interest.
