# Supplementary figures and images for: Circadian regulation of metabolic, cell division, and cation transport promoters in the gastrointestinal bacterium Klebsiella aerogenes
Source: Front Microbiol. 2023 Jul 5;14:1181756. doi: 10.3389/fmicb.2023.1181756 (PMC10356819; doi:10.3389/fmicb.2023.1181756)

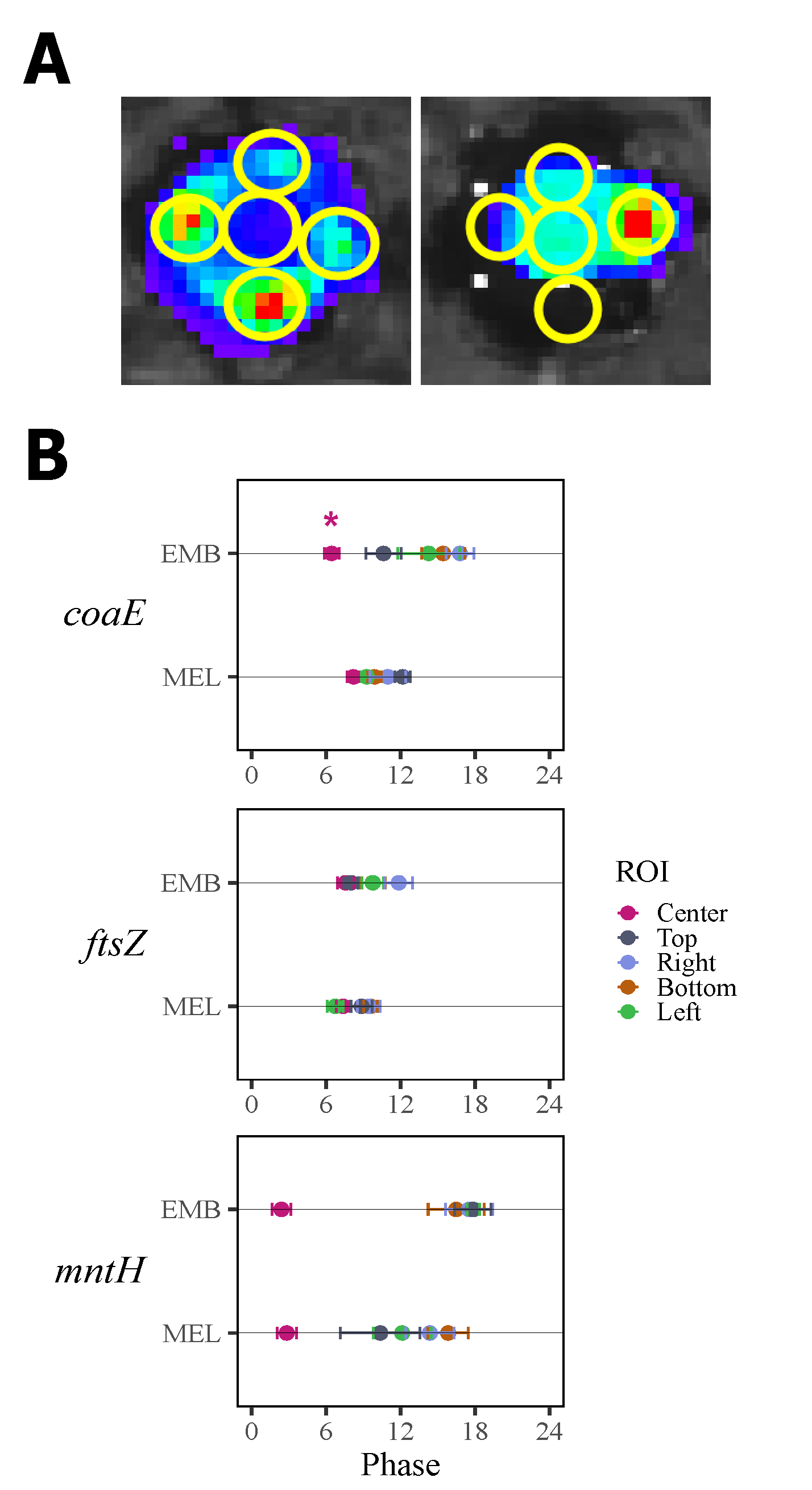

Supplement: Supplementary file 1 [file Image_1.TIFF]

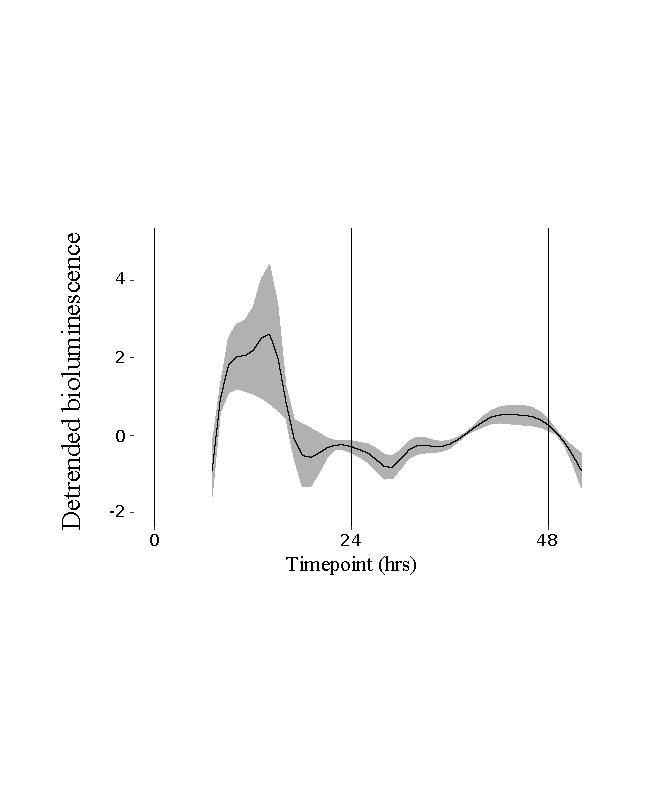

Supplement: Supplementary file 2 [file Image_2.TIFF]
